# Lessons from sexual and reproductive health voucher program design and function: a comprehensive review

**DOI:** 10.1186/1475-9276-13-33

**Published:** 2014-04-29

**Authors:** Corinne Grainger, Anna Gorter, Jerry Okal, Ben Bellows

**Affiliations:** 1 Options Consultancy Services Ltd., Senior Technical Specialist, Devon House, 58 St Katharine’s Way, London E1W 1LB, UK; 2 Instituto CentroAmericano de la Salud, Epidemiology, Managua, Nicaragua; 3 Population Council, Ralph Bunche Rd., PO Box 17643-00500, Nairobi, Kenya

**Keywords:** Results-based financing, Demand-side financing, Sexual and reproductive health, Maternal health, Voucher program, Social franchising, Poverty targeting, Social health insurance, Incentives, Subsidies

## Abstract

**Background:**

Developing countries face challenges in financing healthcare; often the poor do not receive the most basic services. The past decade has seen a sharp increase in the number of voucher programs, which target output-based subsidies for specific services to poor and underserved groups. The dearth of literature that examines lessons learned risks the wheel being endlessly reinvented. This paper examines commonalities and differences in voucher design and implementation, highlighting lessons learned for the design of new voucher programmes.

**Methodology:**

The methodology comprised: discussion among key experts to develop inclusion/exclusion criteria; up-dating the literature database used by the DFID systematic review of voucher programs; and networking with key contacts to identify new programs and obtain additional program documents. We identified 40 programs for review and extracted a dataset of more than 120 program characteristics for detailed analysis.

**Results:**

All programs aimed to increase utilisation of healthcare, particularly maternal health services, overwhelmingly among low-income populations. The majority contract(ed) private providers, or public *and* private providers, and all facilitate(d) access to services that are well defined, time-limited and reflect the country’s stated health priorities.

All voucher programs incorporate a governing body, management agency, contracted providers and target population, and all share the same incentive structure: the transfer of subsidies from consumers to service providers, resulting in a strong effect on both consumer and provider behaviour. Vouchers deliver subsidies to individuals, who in the absence of the subsidy would likely not have sought care, and in all programs a positive behavioural response is observed, with providers investing voucher revenue to attract more clients. A large majority of programs studied used targeting mechanisms.

**Conclusions:**

While many programs remain too small to address national-level need among the poor, large programs are being developed at a rate of one every two years, with further programs in the pipeline. The importance of addressing inequalities in access to basic services is recognized as an important component in the drive to achieve universal health coverage; vouchers are increasingly acknowledged as a promising targeting mechanism in this context, particularly where social health insurance is not yet feasible.

## Introduction

### Dramatic gaps in health

Globalization is a shorthand term for dramatic economic expansion and growing international interdependence among high-income countries and a large set of post-colonial, low-income countries since the 1980s. That convergence also changes the concept of “developing country” as low-income countries cross into the low-middle income bracket. Yet as globalization has pulled millions from poverty, it has also opened a widening equity gap within countries in terms of income and health status. There are particularly large gaps in healthcare access, and often the poor and vulnerable do not receive the most basic of reproductive health services [1].

Current health service provision in many low-income countries does not meet public needs and among the community of aid actors there is frustration with the lack of results achieved by more traditional input-based approaches, such as support for training, infrastructure, drugs and supplies, and behaviour change communication. Many governments are aware of the low performance of their health systems and are ready to test new approaches, particularly those which can target underserved groups with priority health services, such as voucher schemes. The proliferation in the number of voucher schemes since 2005, and the dearth of literature which examines lessons learned from program design and implementation, risks the wheel being endlessly reinvented. This paper examines commonalities and differences in voucher design and implementation, and highlights lessons learned for the design of new voucher programmes, based on a review of 40 programs.

### Result-based financing

During the last two decades, donors and governments have invested in alternative financing models where financial payments and other incentives are linked to outputs. The umbrella term for these approaches is results-based financing (RBF) [2], defined as *"a cash payment or non-monetary transfer made to a national or sub-national government, manager, provider, payer or consumer of health services after predefined results have been attained and verified. Payment is conditional on measurable actions being undertaken*”. RBF includes a wide range of approaches which vary according to, among other things, the objectives, the remunerated behaviours (or indicators), the entity receiving the reward and the type and magnitude of the financial reward. The common denominator in all these strategies is payment, in some form, for results as opposed to exclusively financing inputs.

**Figure 1:**
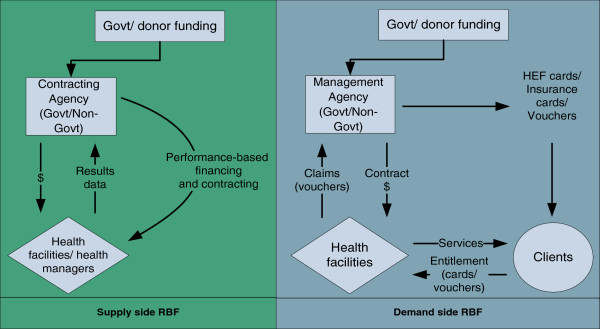
Supply-side and demand-side results-based financing approaches.

A standard categorization is to distinguish RBF schemes that offer incentives on the supply side (supply-side RBF) from those with an incentive structure primarily on the consumer side (demand-side RBF), although in practice the boundary between these categories is not clear cut. This is illustrated in Figure 1 below. In a supply-side RBF approach, incentives are paid to the provider based on results reported on a (set of) performance target(s) or indicator(s). Where incentives are linked to, say, increased utilisation of services by a specific target group, this will have an *indirect* impact on the demand-side as health providers put in place more or less successful measures to reach their targets and earn incentives. In demand-side RBF there is a more *direct* link between the payment of incentives, the actions of the intended beneficiary and the desired result. Vouchers are a demand-side RBF approach with a strong supply-side effect; the behaviour of both provider and consumer is directly influenced by the incentive.

### Voucher schemes

**Figure 2:**
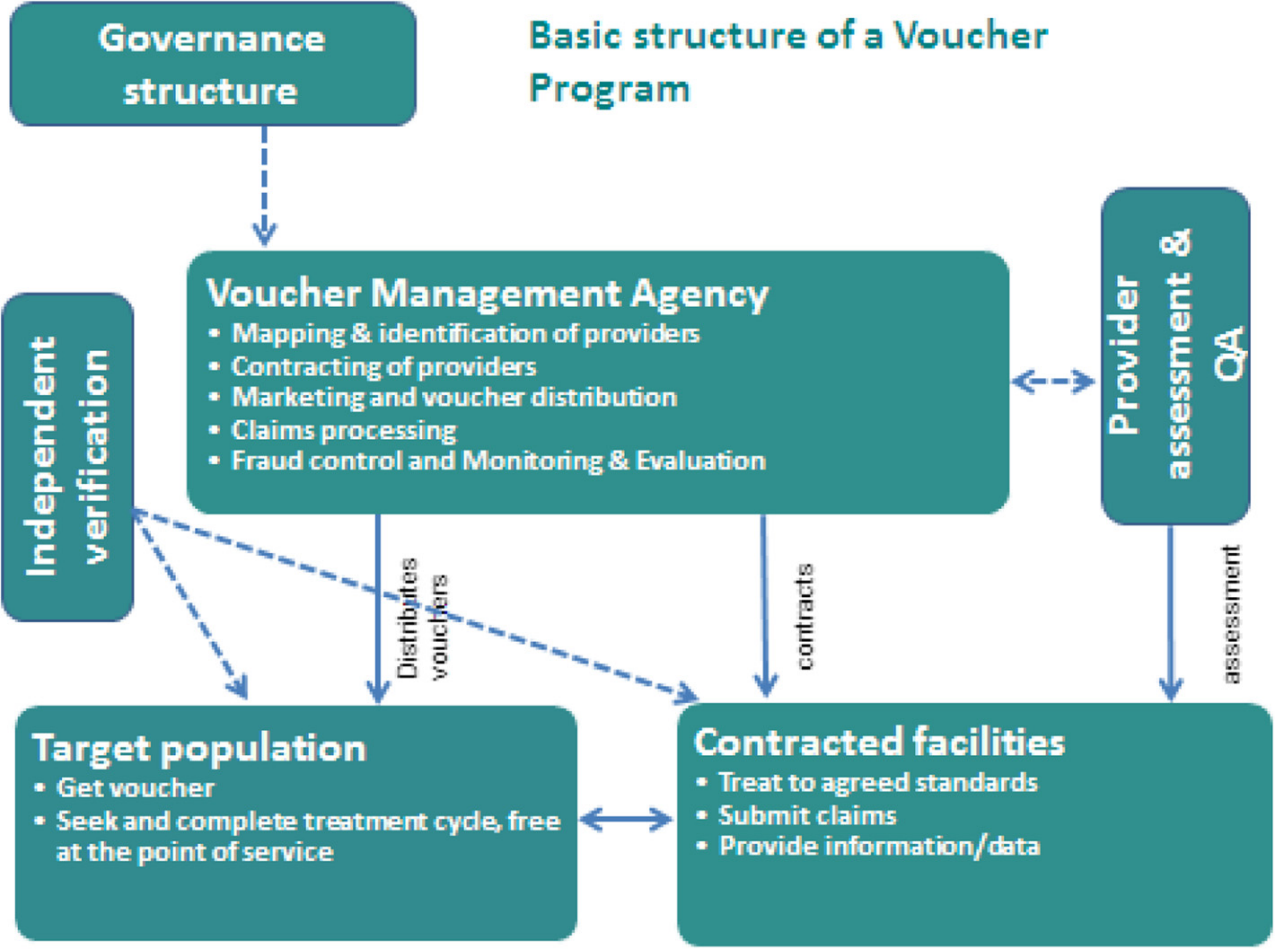
Key characteristics of voucher programs.

Vouchers are commonly used to channel subsidies (from governments and/or donors) to stimulate demand for priority health services among specific underserved groups. Figure 2 illustrates the basic structure of a voucher programme. Subsidies go directly to the consumer in the form of a voucher – a certificate, coupon or other token – which the consumer exchanges for the specified goods or services from an accredited or approved health facility (public or private). The provider then claims payment for services provided. Vouchers are usually competitive with multiple providers; however, they can also be non-competitive, i.e. working with fewer providers of a single type [3]. Most healthcare voucher programs have been designed to increase access to one or more sexual and reproductive health (SRH) services.

Although there are many variations in the design and implementation arrangements of voucher programs, they share a number of important characteristics: a funding body (government and/or donors), a governance structure that oversees the program, and an implementing body (e.g. voucher management agency) that distributes vouchers to target populations, approves and contracts facilities to provide services to voucher clients, and reimburses the facilities for services provided.

Vouchers are proving to be an interesting approach to overcoming barriers related to accessing SRH care for the poor and other vulnerable groups. There is growing evidence that vouchers promote equity in access to specific health services, can offer financial protection and lead to improved quality of care; cornerstones of the move towards universal health coverage. Two recent systematic reviews of the evidence of the impact of voucher programs on a range of variables found robust evidence that vouchers can increase utilization of health services, and modest evidence that voucher programs both improve the quality of service provision and effectively target resources to specific populations [4,5]. Although these results were based on the review of relatively few underlying voucher programmes, newly published and newly discovered studies support these findings, and provide new evidence that vouchers are effective at targeting and enhancing equity [6-11]. There are very few studies of the impact of vouchers on health status or efficiency.

While recent documentation has focused on analysing the potential impact of voucher programs, none of the literature has attempted to draw out lessons learned for the design of new programs. The review by Meyers et al., [5] highlighted the fact that program managers of current and future voucher programs would benefit from a review of lessons learned when implementing voucher programs. This paper presents a timely and comprehensive review of voucher program design and implementation arrangements based on an analysis of documentation on 40 different voucher schemes.

## Methods

The objective of the review was to analyse the design and different implementation arrangements for voucher programs for SRH services.

Through extensive discussion among the group of authors, all of whom are experts on voucher program design and evaluation, we developed the following inclusion and exclusion criteria for the review:

● The inclusion of all voucher programs for health services which started distribution of vouchers before 28 February 2011. The cut-off date for the review was June 2011 and a period of at least three months of operation was considered necessary in order to look at the functioning of a particular program;

● The inclusion of voucher programs *which do not use a physical voucher*, but which function in all other respects as a voucher program (e.g. targeting the poor through the use of Below Poverty Line cards in India);

● The exclusion of programs that use vouchers for goods (condoms, pills, insecticide treated bed nets to prevent malaria) as opposed to services. Design and implementation arrangements differ considerably between voucher programs for goods and voucher programs for services. Voucher programs for goods function more like social marketing programs;

● The exclusion of those voucher programs that are operating in high income countries^a^;

● The exclusion of programs where there is no reimbursement to the facility or provider. These include programs where a voucher is only used as a marketing tool to attract clients to a facility, where vouchers are used for referral services between health facilities only, or where vouchers are used for research (tracking of clients, data collection, etc.). It also excludes conditional cash transfer programs where there is no provider reimbursement payment since these are pure demand-side programs which do not provide incentives (and therefore do not channel funding) to health service providers as in voucher schemes.

Using the above inclusion and exclusion criteria, we conducted a comprehensive review and compiled a list of all voucher programs. The literature database developed through the DFID systematic review and which included data up to October 2010 was used as the basis [5]. We then used the same methodology to update this database from April to December 2011 with: (i) searches of bibliographic databases using specified key words (i.e. voucher, coupon, certificate); (ii) a review by hand of the grey literature; (iii) back checking of references for all selected articles and documents: (iv) checking of organisational networks and websites, as well as (v) extensive networking and sourced information from key contacts.

The aforementioned review by Bellows et al. (2011) identified 13 voucher programs, all providing SRH services in developing countries. The review by Meyer et al. (2011) identified 43 voucher programs, including the 13 programs of Bellows et al. and also including voucher programs for goods (e.g. insecticide treated bed nets) [4,5]. Of the 43, a total of 21 programs fit the criteria for our comprehensive review, which also identified 19 additional programs giving a total of 40 programs.

The database on these 40 identified voucher programmes was then enhanced through additional searches in order to obtain more detailed information related to context, design and implementation arrangements. Networking and correspondence with key contacts was particularly useful in identifying new programs and in providing program descriptions (e.g. reports), tools (e.g. contracts, operational manuals, vouchers), and other relevant material. A list of published documents consulted, organised by country, is included as Appendix.

We developed a list of 120 program characteristics, which were thought to be relevant for the design and implementation of voucher programs through extensive discussions with voucher experts. The characteristics were extracted from the literature, collected and input into an excel database to facilitate analysis, for example summing (e.g. type of voucher service, type of health service provider), defining averages (e.g. available budget), cross analysis (e.g. type of management agency against type of service providers contracted) and so on. These characteristics were then grouped into five major categories related to design and implementation of the voucher programs:

1. General aspects: size and geographical coverage, statement of objectives, timeframe and financing;

2. Management and governance: structural aspects of voucher programs such as governance, implementing and/or managing entity and its relation to contracted providers; role and participation of government;

3. Benefits and targeting: benefit and client policies such as services covered, distribution strategies (i.e. sold or freely distributed), and targeting approaches;

4. Providers: types of provider; competition; selection and contracting; price of services and reimbursement to providers;

5. Implementation arrangements: marketing, training, voucher distribution, claims processing, monitoring and evaluation, and fraud control.

In this paper, we describe the results of the analysis of these five categories, looking at commonalities and differences and identifying the lessons learned for programmers.

This systematic review of the literature relies on secondary published and unpublished literature. Ethics review was therefore not necessary.

## Results

### General program characteristics: objectives, financing, size and coverage

Forty voucher programs were identified in this comprehensive review (see Table 1); 22 are still active and 18 ceased to exist. Of the 18 programs that have ended, five programs met their original objectives; five were studies or pilots either taken over by or informing new programs; one program was incorporated into a Health Equity Fund; and seven programs were unable to find new funding, most of them belonging to the older programs developed during the 1990s.

**Table 1:** Details of the 40 identified voucher programs

	**Country**	**Initiated by**	**Years**^**1**^	**Reason to use vouchers**	**Services**	**Type providers**	**Type VMA**	**Size VP**^**2**^
**1**	**Armenia**^**3**^	**Government**	**2008-ong**^**4**^**.**	**Curb informal payments**	**SMH, CD**^**5**^	**Public (few), private**	**Government**	**Large**
**2**	**Bangladesh 1**	**Government/donor SWAp**	**2006-ong.**	**Increase use priority services**	**SMH**	**All three, most public**	**Gov./WHO**	**Large**
3	Bangladesh 2	Research center - ICDDR,B	2006-2008	Op Research to test vouchers for skilled birth attendance	SMH	Only private	University	Small
4	Bangladesh 3	Intern. NGO – Pop Council	2007-2008	Op Research to test vouchers to improve up-take of MNCH services among poor rural women	SMH	All three sectors	NGO	Small
5	Bangladesh 4	Social Franchise - MSI	2007-2010	Increase use priority services	SMH	All three sectors	SFO	Small
6	Cambodia 1	Donor - BTC	2007-2010	Expand HEF to Health Centers	SMH	Only public	NGO	Small
7	Cambodia 2	UN organization - UNFPA	2008-2010	Expand HEF to Health Centers	SMH, FP, SA, STI	Only public	NGO	Small
**8**	**Cambodia 3**	**Donor - USAID**	**2009-ong.**	**Expand HEF to Health Centers**	**SMH**	**Only public**	**NGO**	**Small**
**9**	**Cambodia 4**	**Donor - KfW**	**2011-ong.**	**Introduce social health insurance skills**	**SMH, FP, SA**	**All three sectors**	**Private/NGO**	**Large**
**10**	**Cambodia 5**	**Social Franchise - MSI**	**2010-ong.**	**Increase use at trained facilities**	**FP**	**All three sectors**	**SFO**	**Small**
11	China 1	Government/World Bank	1998-2001	Increase use priority services	SMH, CD	Only public	Gov./Project	Medium
12	China 2	Government/World Bank	2005-2007	Increase use priority services	SMH, RTIs	Only public	Gov./Project	Small
**13**	**India-Agra, UP**	**Donor - USAID/State Gov.**	**2007-ong.**	**Contract private sector/build PPP**	**SMH, FP, STI/RTI**	**only private**	**Government**	**Small**
**14**	**India-Kanpur, UP**	**Donor - USAID/State Gov.**	**2008-ong.**	**Contract private sector/build PPP**	**SMH, FP, STI/RTI**	**NGO and private**	**NGO**	**Medium**
**15**	**India-Jharkhand**	**Donor - USAID**	**2009-2011**	**Contract private sector/build PPP**	**FP**	**Only private**	**NGO**	**Small**
**16**	**India-Uttarakhand**	**Donor - USAID/State Gov.**	**2007-ong.**	**Contract private sector/build PPP**	**SMH, FP**	**NGO and private**	**Government**	**Medium**
**17**	**India-Gujarat**	**State Government**	**2005-ong.**	**Contract private sector/limited public capacity**	**SMH**	**Only private**	**Government**	**Large**
18	India-Rajastan	Local NGO	2003-2006	Contract private sector/limited public capacity	SMH	Only private	NGO	Small
19	India-Kolkata	Donor (Gates)/NGO	1999-2003	Contract private sector/limited public capacity	SMH, FP, STI/RTI, CD	Only private	NGO	Small
**20**	**India-Delhi**	**State Government**	**2008-ong.**	**Contract private sector/limited public capacity**	**SMH**	**Only private**	**Government**	**Medium**
**21**	**India-Haryana**	**State Government**	**2006-2011**	**Contract private sector/limited public capacity**	**SMH**	**Only private**	**NGO**	**Small**
22	Indonesia	Government/World Bank	1998-2004	Contract private sector/limited public capacity	SMH, FP	Only private	Gov./Project	Medium
**23**	**Kenya 1**	**Donor - KfW**	**2006-ong.**	**Introduce social health insurance skills**	**SMH, FP, GBV**	**All three sectors**	**Private**	**Large**
24	Kenya 2	Intern. NGO - Popcouncil	1997-2010	Contract private sector/preference of target population	SRH care for youth	Public (few), private	NGO	Small
25	Korea	Government	1964- ~1985	Contract private sector/facilitate M&E	FP	Public (few), private	Government	Large
**26**	**Madagascar**	**Social Franchise - PSI**	**2005-ong.**	**Increase use by poor at franchise clinics**	**SRH care for youth**	**Only private (SF)**	**SFO**	**Small**
**27**	**Myanmar**	**Social Franchise - PSI**	**2005-ong.**	**Increase use by poor at franchise clinics**	**FP, STIs**	**Only private (SF)**	**SFO**	**Small**
28	Nicaragua-Sex Workers	Local NGO	1996-2009	Contract private sector/preference of target population	STIs	All three sectors	NGO	Small
29	Nicaragua-adolescents	Local NGO	2000-2005	Contract private sector/preference of target population	SRH care for youth	All three sectors	NGO	Small
30	Nicaragua-Cervical Cancer	Local NGO	1999-2009	Contract private sector/preference of target population	Cervical Cancer scr.	All three sectors	NGO	Small
**31**	**Pakistan (MSI)**	**Social Franchise - MSI**	**2008-ong.**	**Increase use by poor at franchise clinics**	**FP**	**Only private (SF)**	**SFO**	**Small**
32	Pakistan-DG Khan	Social Franchise - PSI	2008-2009	Increase use by poor at franchise clinics	SMH	Only private (SF)	SFO	Small
**33**	**Pakistan-Jhang**	**Social Franchise - PSI**	**2009-ong.**	**Increase use by poor at franchise clinics**	**SMH**	**Public (few), private**	**SFO**	**Small**
**34**	**Pakistan-Charsadda**	**Donor-KfW (PSI implemented)**	**2010-2011**	**Introduce social health insurance skills**	**SMH**	**Public (few), private**	**SFO**	**Small**
**35**	**Pakistan-Sehat Sahulat Card**	**Local Government**	**2009-ong.**	**Contract private sector/limited public capacity**	**SMH**	**public (few), private**	**Private**	**Small**
**36**	**Sierra Leone**	**Social Franchise - MSI**	**2009-ong.**	**Increase use by poor at franchise clinics**	**SMH, FP**	**NGO and private**	**SFO**	**Small**
37	Taiwan	Government	1964- ~1985	Contract private sector/facilitate M&E	FP	Public (few), private	Government	Large
**38**	**Uganda (KfW/GPOBA)**	**Donor-KfW and GPOBA (MSI implemented)**	**2006-ong.**	**Introduce social health insurance skills**	**STIs, SMH, FP**	**NGO and private**	**SFO**	**Large**
**39**	**Uganda (University)**	**Makerere University**	**2009-2011**	**Research study to assess vouchers for Institutional Delivery & transport**	**SMH**	**All three sectors**	**University**	**Small**
40	Vietnam-Sex Workers	Int. NGO-Pathfinder	2009-2009	Contract Private Sector/preference of target population	STI	Only private	Government	Small

Notes: ^1^Voucher Programmes that are active up to December 2011; ^2^Size VP indicates the annual budget in three categories: large (greater than $1 million), medium ($250,000 to $1 million), and small (less than $250,000); ^3^Rows in bold are active programs; ^4^Ong (on-going) = continued into 2012; ^5^CD = Childhood diseases.

A review of voucher program objectives generated a list of reasons for choosing vouchers over an alternative approach. Nearly all programs address a combination of objectives with the most common being: increasing access to priority services among underserved and vulnerable populations; accelerating the use of underutilized services; and expanding provision of priority services through contracting of private sector facilities (e.g. in countries where most providers are private or where there are large gaps in public service provision). Introducing choice for clients and competition between service providers to drive quality improvements; and increasing transparency and verification of service delivery are other secondary objectives mentioned in the literature. An overriding and implicit goal of many voucher programs is that of preventing catastrophic out-of-pocket payments for healthcare among the poor. This is particularly relevant for maternal and newborn health care where the potential treatment costs are unknown when a woman arrives to deliver, and can be very high for complicated cases.

The review also shows that vouchers can be used to curb informal payments (e.g. Armenia) or to introduce social health insurance capacity into the health sector (e.g. voucher programs financed by the German Development Bank, KfW, in Cambodia, Kenya, Tanzania and Uganda). The longer-term stated objectives for the KfW-financed voucher schemes is that, by introducing skills that are relevant to social health insurance, vouchers will help governments to develop their capacity to purchase health services (accreditation, pricing, contracting, quality assurance, monitoring, claims processing and reimbursement) and to target subsidies to particular needy populations. This is also true of voucher programs designed after the cut-off date for our study, such as a maternal and newborn health voucher scheme in Yemen being designed with support from the World Bank, which explicitly supports a move towards the separation of the roles of ‘purchaser’ and ‘provider’ of health services. This is being achieved through capacity building of a semi-autonomous voucher management agency as the purchaser of a defined package of maternal, newborn and reproductive health services from both public and private sectors. The extent to which voucher schemes are able to build this capacity with host-country governments needs to be closely monitored.

The authors did not find reference in the literature to voucher programs where the original objectives of a program were substantially changed. What is clear is that voucher programs can and do adapt to changes in the external operating environment, such as changes in policies on user fees, levels of provider autonomy, willingness of the government to contract with private providers and so on. There is also evidence that voucher schemes can be adapted (and often expanded) to incorporate lessons learned, as the success or failure of particular strategies becomes clearer, and new funding agencies are attracted with their own agendas. This is well illustrated by the progression of the voucher program in Uganda (See *Uganda Case Study below*).

***Uganda Case Study:*** Following a feasibility study in 2004, the KfW-financed Uganda voucher program issued its first voucher providing access to STI diagnosis and treatment (the Healthy Life voucher) in 2006. In 2008, with joint funding by KfW and GPOBA, a safe motherhood voucher was added (Healthy Baby voucher) and the scheme was expanded to become the Reproductive Health Voucher Program (RHVP). While the STI voucher was effectively available to everyone via selected pharmacies located in poorer socio-economic areas, the Healthy Baby voucher was explicitly targeted at poorer clients identified through door-to-door visits using a poverty assessment tool. In 2011, with funding from USAID and DFID a family planning voucher was added through a new scheme (Saving Mothers, Giving Life) which used the same systems and processes set up for RHVP, but is piloting a transport voucher, and expanded BCC activities to include nutrition. Whereas to date the voucher schemes have all worked exclusively with private providers (commercial and not-for-profit), the government is currently working with the World Bank to scale-up the voucher approach nationally using a mix of public and private providers and providing access to an expanded basket of services.

The majority of the voucher programs reviewed (see Table 2) are in Asia (31 out of 40). In Asia, India has (or has had) nine voucher programs, followed by Pakistan (5), Cambodia (5) and Bangladesh (4).

**Table 2:** Number of voucher programs in each region and country

**Regions**	**Voucher programs**	**Countries**
Latin America	3	Nicaragua (3)
Africa	6	Kenya (2), Uganda (2), Sierra Leone, Madagascar
Asia	31	
• West Asia	1	Armenia
• South Asia	18	India (9), Pakistan (5), Bangladesh (4)
• East Asia and Pacific	12	Cambodia (5), China (2), Indonesia, Korea, Myanmar, Taiwan, Vietnam
All	40	

Over a quarter of the programs were initiated by a donor, often directly engaging with government, and mostly in Asia (see Table 3). Governments, including state governments in India, initiated eleven programs, four in close collaboration with donors. Interestingly all of the government-initiated programs are in Asia. Outside Asia, the two programs classed as large were initiated by the German Development Bank (KfW) in Kenya and Uganda, and the rest are small voucher programs that have been started by social franchising organizations, Non-Governmental Organizations (NGOs) and research institutes, nearly always with donor support.

**Table 3:** Type of organization that initiated the voucher program

**Initiated by**	**Voucher programs**	**Observation**
Donor	11	9 in Asia and 2 in Africa
Government	11	All in Asia, 4 in collaboration with donors
SFO	8	6 in Asia, 2 in Africa
NGO	7	3 in Asia, 1 in Africa, 3 in Latin America
Research institute	2	1 in Asia (Bangladesh), 1 in Africa (Uganda)
UNFPA	1	1 in Asia (Cambodia)
All	40	

**Figure 3:**
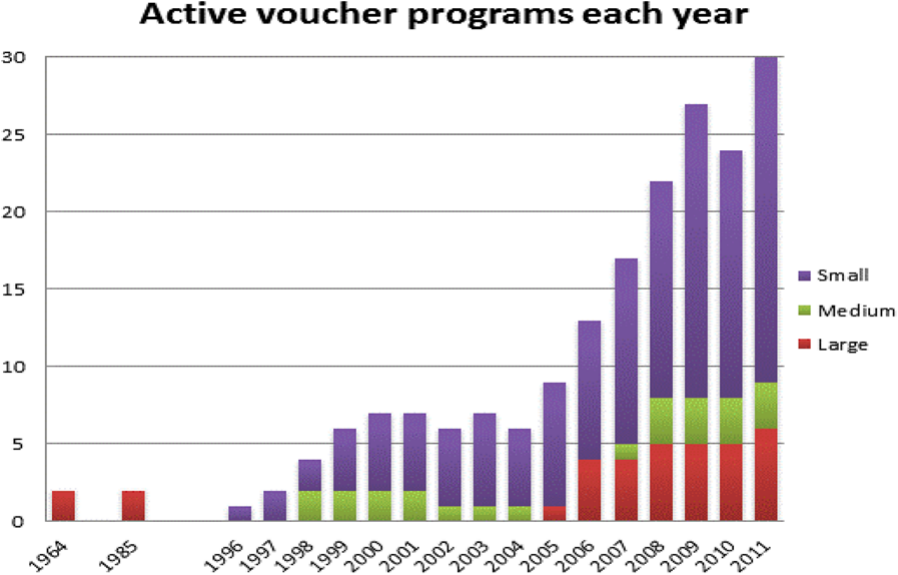
Number of active voucher programs in each year 1964–2011.

Figure 3 shows the number of voucher programs that were active in a particular year. The figure illustrates clearly the huge increase in the number of voucher programs, particularly since 2004. The first two schemes were developed in 1964 (Taiwan and Korea) with the objective to lower the fertility rate through accelerating the use of family planning. After a small pilot in each country, the voucher programs were quickly scaled nationwide and continued until the mid-1980s when fertility had reached replacement level.

Between the late 1990s and 2004 only a handful of programs were initiated (six small programs and two medium-sized projects in China and Indonesia), coinciding with a period when bilateral aid was firmly focused on direct support to governments. In 2005, the number of voucher programs began to increase and Social Franchising Organizations also started to introduce vouchers to increase use of SRH services by poor and disadvantaged populations at franchised clinics, accounting for a significant part of the more recent increase in voucher schemes. The number of active voucher programs in 2011 (Figure 3) includes eight voucher programs that were initiated after the cut-off date of this review.

Eight of the voucher programs can be categorized as ‘large’ in size with a budget of over US$1 million per annum (see also Figure 3). Large voucher programs that are on-going at the time of writing include the KfW-funded voucher programs in Cambodia, Kenya and Uganda, the Armenian Obstetric Care State Certificate (OCSC) program, the Indian scheme in Gujarat, and the large voucher program in Bangladesh (also known as the Demand Side Financing or ‘DSF program’). The earlier programs in Taiwan and Korea also had very large budgets that varied from year to year and were national in scope.

Only three voucher programs have been implemented nationally: Armenia, Taiwan and Korea. The voucher program in Gujarat, India, which targets the population with a Below-Poverty-Line or BPL card, is implemented state-wide and, with a population of some 60 million people, Gujarat is larger than Armenia, Taiwan or Korea. An estimated budget of some US$7 m per year also means that this has been one of the largest voucher programs. The other programs identified as ‘large’ in this review cover only a fraction of the general population: the DSF program in Bangladesh covers around 10% of upazilas or sub-districts, and the large KfW-funded voucher programs in Cambodia, Kenya and Uganda target the poor, delivering approximately 3% of all births nationally and operating in between five and 20 districts depending, among other things, on how long they have been in operation. The World Bank is currently supporting the Government of Uganda in the design of a nationally scaled voucher program (see Uganda case study) with multiple funding sources (mostly multilateral and bilateral donors), as well as a further large voucher program in the Republic of Yemen.

Four voucher programs are of medium size (budgets of between US$250,000 and US$1 million per year), and nearly three quarters (28) of the programs are small, with budgets of less than US$250,000 per year, reflecting both the number of pilot interventions and the recent proliferation of small but growing voucher programs attached to social franchise networks. These have been introduced by franchising organisations, such as Marie Stopes International (MSI) or Population Services International (PSI), with the aim of growing the market for first time service users (who may become paying clients over time) and of meeting equity-related objectives and conditions, often attached to donor funding.

### Management and governance

There are different management structures among the programs reviewed, largely due to the type of initiating agency (i.e. whether government or non-government), and the need for programs to be tailored to the context in which they are designed (e.g. type of providers available or willingness of government to work with the private sector).

Programs initiated by donors are sometimes managed by the government, but mostly by a private agency (profit or not-for-profit) as the so-called voucher management agency, which is responsible for implementation (identifying, contracting and monitoring providers, distributing vouchers to intended beneficiaries, and organising payments for verified service delivery). Most managing agencies are assigned; only in two cases has this function been tendered, e.g. in the KfW-funded programs in Kenya and Cambodia where this was related to donor procurement rules. The governing body, which oversees the program, is mostly a steering committee or project advisory group, with representatives from government, donors and other stakeholders, often fully independent from the managing agency.

In government-initiated programs, the agencies responsible for implementation and governance are often both from the public sector (i.e. Ministry of Health). In these situations, the governing body is often at central or provincial level while the management function is at lower levels. For example, in Gujarat the State Health Directorate oversees the program while project management units at the district level are implementing the program and also act as the managing agency.

Programs initiated by NGOs or social franchising organisations and research institutes, which are mostly financed by donors, are all managed by the organizations themselves. Hence the governance structure is also the same as the managing agency. In four of the ten voucher schemes managed by social franchising organisations, providers are restricted to the franchise network, which could limit competition and lead to gaps in coverage.

The argument for a strong governance structure gets more compelling as the funding for a voucher program increases and the program expands, with the attendant opportunities for fraud. As stewards of the health sector, it is important that the government has oversight of any large health financing intervention, particularly those that target the poor. This has been seen in both Kenya and Cambodia where management structures have been adjusted to articulate more clearly the responsibility of government in overseeing the voucher schemes (see Governance Case Study, Kenya below).

While the potential for fraud is an often-cited concern for donors, a strong management information system (MIS) and a robust claims processing system, verification of results (often by an independent agency), monitoring and enforcement of annual contracts with providers, and strong checks and balances employed by the managing agency, may account for the relatively low incidence of fraud reported in the literature (although fraud may well be under-reported). The most common types of fraud encountered include: providers purchasing vouchers and seeking reimbursement for fictitious clients; distributors or clients forming an alliance with providers without provision of actual services; providers handing in false claims; service providers inflate complications treated and claim for higher amounts; and the providers charging additional fees from voucher holders.

Those voucher schemes managed by a third party managing agency, such as the large KfW-financed schemes in Kenya and Cambodia, have strong anti-fraud protection measures built into the design, based on a twin strategy of analysing trends in voucher distribution and claims made, and on verifying samples of claims (randomly generated by the program management information system) at the level of the voucher service provider and at the beneficiary’s home. Knowledge of ‘what works’ in fraud protection is being built into the design of new voucher schemes (i.e. in Yemen and Moçambique).

Other common checks and balances reported to counteract fraud include the use of unique serial numbers, and use of spot checks. These mechanisms, if employed carefully, will counteract all types of fraud listed above. It should also be remembered that, even though fraud is notoriously difficult to quantify, all health systems, however the financing of services is organized, experience a degree of fraud [11].

In all large programs, contracts enable the managing agency to exclude providers from the program or to enact other sanctions for fraudulent behaviour (e.g. the KfW-financed programs in Cambodia, Kenya and Uganda, and the national scheme in Armenia). Where contracts are not well enforced, stakeholders are able to adapt the scheme to suit their own needs such as in the Chiranjeevi Scheme in Gujarat where a flat reimbursement for every 100 deliveries regardless of the number of C-sections or complicated cases led to private obstetricians referring complicated cases to public sector facilities in order to reduce costs.

Independence and autonomy of the managing agency has often been cited as an important feature for the ‘ideal’ governance and management structure to prevent fraud (because the body overseeing the program can independently sub-contract quality assurance or verification and has more leverage over the managing agency than when both bodies are from the same organisation). However, our review shows that both forms are functional among the voucher programs analysed and none of the literature examined the effect of independent governance structures on the functioning of the program in terms of reducing fraud or increasing the quality of care provided.

Perhaps more important is the split between purchaser and provider; that is the managing agency contracts providers not belonging to its own organisation, in order to ensure transparency, widen choice, increase efficiency and counteract fraud. Although in nearly all voucher programs there is a split between purchaser and provider (i.e. in 37 of the 40 programs analysed the managing agency contracted providers from a different organisation and/or sector), there are also exceptions such as the large voucher program in Bangladesh and two programs in China where the managing agency and large majority of providers belong to the public sector, but which nonetheless functioned well. However, in these programs, there are still checks and balances in place that provide a degree of management independence (in Bangladesh this results from a special programme management unit set up for management of the scheme, and in China the WHO financed supervisors within the state voucher management unit).

***Governance Case Study, Kenya:*** Initiated in 2005, the KfW-financed project in Kenya (RH-OBA project) will enter its fourth phase in 2014. The Government of Kenya (GoK) has begun to take on a more significant role with regard to both financial contributions to, and governance of, the program. In 2011, the oversight of the program moved from the National Coordinating Agency for Population and Development, a semi-autonomous agency, to the Ministry of Public Health and Sanitation (MoPHS), which has since merged with the Ministry of Medical Services (MoMS). Other key changes have included constituting a Program Management Unit (PMU) in government and establishing linkages with sub-national health management systems, with the aim of building sustainability. The new State Department of Health, the National Hospital Insurance Fund (NHIF) and KfW together sit on the Steering Committee. Throughout this time, the managing agency contract has remained with PricewaterhouseCoopers, responsible for the operational management of the scheme. During Phase III, the GoK has increased its contributions to the program, and significant efforts will be made in Phase IV to further build the institutional and technical capacity of the PMU in health financing and to adapt the design of the voucher scheme to the 2013 policy on free maternal health services.

### Provider autonomy

The issue of provider autonomy is not explored in detail in the literature. It is clear that the optimum model is for providers to have autonomy, not only to reinvest voucher payments on quality improvements, staff incentives and other activities but also to organise and manage services so as to attract clients in the target group more efficiently and effectively. While provider autonomy is the norm when private providers are contracted into voucher programs, there are varying degrees of autonomy with participating public and also not-for-profit providers. No public voucher service provider reported having full autonomy to organise service provision according to the requirements of the voucher program, i.e. including the ability to hire and fire medical staff. Voucher programs can provide a framework for moving towards greater provider autonomy, at least in terms of reinvesting voucher income, and this has been seen in the case of the KfW-financed voucher program in Kenya where some public sector voucher service providers have been able to invest a growing proportion of their voucher income in improving service quality, due to the support and influence of the managing agency.

### Voucher benefits and clients (demand side characteristics)

Currently voucher programs provide access to only a limited ‘basket’ of services, with 26 out of 40 programs providing only one type and 7 providing two types of service – often a combination of safe motherhood (SMH) and Family Planning (FP) services. It is worth noting, however, that one ‘type’ of service may in fact be a package of safe motherhood services (including antenatal and postnatal care, normal and complicated deliveries, and post-natal FP) or in the case of programs in Armenia and China a package of child health services.

**Figure 4:**
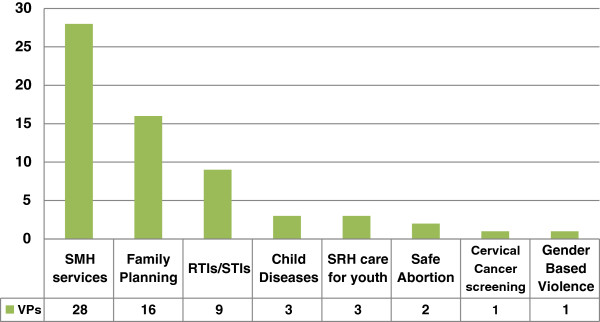
Type of services provided and number of programs providing that particular service (of 40 programs reviewed).

A further 7 programs have expanded to provide three or four different service types, reflecting on-going discussions about expanding access to a broader range of voucher services. Two-thirds of voucher programs provide SMH services, and almost half provide FP services (Figure 4). Other types of service provided through vouchers include diagnosis and treatment of sexually transmitted infections, child health, sexual and reproductive services for young people, safe abortion, cervical cancer screening and gender-based violence recovery services. As voucher schemes introduce more services they begin to resemble insurance schemes. In Tanzania, vouchers are used to enrol (and subsidise) poor pregnant women in a temporary health insurance providing access to a broad package of services, while their families receive subsidised entry to community health insurance, illustrating further the link between vouchers and insurance schemes.

All on-going programs provide free access to services at the point of delivery and the majority also distribute the vouchers for free. Only the KfW-funded programs in Uganda and Kenya and the social franchising programs charge a nominal fee for the voucher.

Once established, voucher programs can be used as a conduit for additional non-medical benefits such as transport, food and cash. Some 10 programs paid for transport costs in addition to the cost of health services, almost all of them in Asia where the cost of transport is a significant barrier to the uptake of health services (Bangladesh, Cambodia, India and Pakistan). The only exception is a small voucher program in Uganda implemented by the University of Makerere which researched the effect of transport vouchers combined with SMH vouchers, and found that the transport vouchers were largely responsible for the huge increases in the utilization of SMH services [12]. It is interesting to note that in the KfW-financed scheme in Kenya, some providers in rural areas pay for transport to the facility out of voucher reimbursement income, demonstrating the importance of transport costs as an access barrier.

Of the on-going voucher programs, nearly all use some form of targeting to channel resources to a priority group (see Table 4). They do this through a combination of mechanisms, most commonly a poverty assessment tool in the form of a questionnaire, a pre-existing poverty identification system such as those used in India (the BPL card) or Cambodia (the Poor ID Card) or geographical targeting of areas identified as ‘poor’. The one current exception is the national program in Armenia, which is untargeted and uses the voucher approach to provide access to priority services for the whole population through a public private partnership framework, similar to the early programs in Korea and Taiwan. Debate is on-going about the relative benefits of means testing, which can be expensive and time consuming and geographical targeting, which is less accurate but with much lower costs [13].

**Table 4:** Targeting characteristics of the 40 voucher programs

**Targeting mechanisms**	**Yes**	**Observation**
Using means testing (MT) with or without other forms of targeting	23	
• Use only means testing (MT)	18	5 VPs in India use a BPL card, 3 in Cambodia use a poor card, others mainly use questionnaires, but 2 VPs in China used community-based participatory approaches to identify the poor
• Use MT in combination with geographical targeting (GT)	3	GT usually used to identify poor rural or slum areas, questionnaires (MT) in urban or peri-urban areas
• MT for SMH and FP services and universal targeting for Safe Abortion and GBV	2	The KfW funded voucher programs used universal targeting for specific services: Cambodia (safe abortion), Kenya (GBV services) and MT for others
Using only geographical targeting	14	A range of VPs in many countries targeted at areas identified as poor such as rural areas (i.e. Nicaragua) or slums (i.e. India) or vulnerable groups in poor areas (adolescents, sex workers)
Using universal targeting	3	Armenia, Taiwan, Korea (Taiwan and Korea moved to MT at a later stage)

The voucher program in Taiwan illustrates the flexibility of the approach in adapting to changes in the operating and policy environment. By altering a few aspects of the program, the government could continue to channel resources to priority services, but in the context of rising program costs and decreasing resources, the voucher moved from universal targeting to a system where the subsidy was targeted only to those less able to pay. The subsidy was reduced or even stopped for the better-off, without major effects on program performance [14].

Vouchers have a marketing and health education function that is relevant in contexts where a change of health seeking behaviour is sought because populations are unfamiliar with, or reluctant to use specific services. Vouchers provide information through marketing and voucher distribution strategies on why the services are important and where to find them, actively inviting clients to use the service. The marketing function of vouchers has been found to be a key aspect of voucher programing, particularly where vouchers are distributed door-to-door or through community meetings, enabling distributors to talk to prospective clients and answer questions (i.e. the KfW-funded voucher schemes in Cambodia, Kenya and Uganda, and the vouchers attached to SF schemes managed by MSI and PSI in Pakistan). Local launch events and community meetings, posters and pamphlets distributed by the managing agency are some of the marketing activities seen in the literature and we also found mention of 8 voucher schemes that used radio and/or TV mostly focused on the launch of a program.

While most programs use a physical voucher, i.e. a booklet or coupon, some make use of existing poverty identification schemes (see above) and two use ‘virtual’ vouchers where services which may be stigmatizing such as gender based violence recovery or abortion services are marketed to the beneficiary group but the vouchers are maintained at the provider and used for claims processing as usual. The paper voucher has a number of functions including providing information about the service and where it can be obtained; acting as evidence for the client and for the clinic that he or she has the right to receive the services for free, thereby helping to prevent informal payments; and acting as a data collection form and paper trail for monitoring and evaluation. It is believed that the voucher itself acts as a sort of personal invitation, empowering its holder to access the services, and this aspect of voucher programming would benefit from research.

Although this was not the case among programs in the current review, there is now a growing interest in using electronic or e-vouchers that use SMS codes sent by mobile phone or other handheld device (i.e. MSI in Madagascar and Ethiopia, Chamganka in Kenya). With mobile phone penetration rates rising and already close to 90 % in some low-income countries [15], the use of e-vouchers will doubtless increase.

### Providers (supply side characteristics)

**Figure 5:**
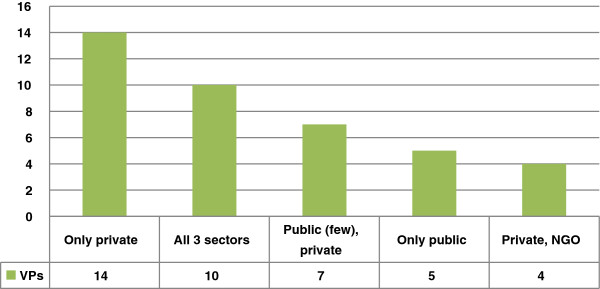
Type of providers in the 40 voucher programs (VP).

Providers participating in voucher schemes come from different sectors: private, NGO (including facilities managed by faith based organizations or social franchising organisations) and public sector. In 14 voucher programs only private sector facilities participated (Figure 5) and half of these are in India where vouchers are routinely used to fill gaps in public provision of priority health services, such as safe and institutional deliveries.

The optimum number of providers in a voucher program should be the number of providers that will both ensure quality of service provision and access to services for a target population, while enabling providers to earn sufficient income through increased client load to attract and keep them in the program.

Of the 40 schemes analysed, there are ten voucher programs that contract or have contracted facilities from all three sectors (public, NGO, and private). Seven programs provide services through a combination of public and private-for-profit providers, although the actual number and role of public providers in these programs is limited due to the lack of available public sector facilities e.g. the large, countrywide programs in Armenia, Korea and Taiwan). Government policies and strategies influence the selection of providers and in five voucher programs only public service providers were contracted, due to government reluctance to contract other types of provider (e.g. Cambodia) and/or because private providers were not available (e.g. China). Four voucher programs contract a combination of NGO and private facilities: two managed by Marie Stopes (Uganda and Sierra Leone) and two funded by USAID in India.

Providers are usually selected according to compliance with minimum quality standards and/or location. Data were incomplete for this variable, but the authors found that documentation for around half of the 40 programs referred to provider selection based on location (i.e. vicinity to a slum) and/or quality (i.e. signal functions). Vouchers are increasingly recognized as presenting an opportunity to introduce and improve accreditation processes and to assist in developing capacity both to measure and maintain the quality of health services, as evidenced in two new voucher programs in Yemen, which are aligned with government quality assessment and assurance procedures.

All voucher programs sign contracts or develop Memoranda of Understanding (MOU) with providers and these agreements should form the basis for monitoring, fraud control and quality assurance. Contracts typically include a description of the services to be provided, the payment schedules and issues related to monitoring, evaluation, fraud control and disputes. Medical protocols and quality criteria are also often included. With the exception of the programs in Nicaragua, the authors found many references in the literature to poor contracting (i.e. lack of detail) and poor enforcement of contracts, which lead to less than optimal performance and quality of service provision.

Successful contracting with private providers can increase the MOH’s interest, enhance local ownership, and also stimulate public providers to raise the quality and client-friendliness of their services. This is the case in Uganda (see Uganda Case Study above), where after six years of contracting with only the private sector, the government is currently considering how to take the voucher program to scale nationally with a mix of public and private providers.

The role of competition in voucher programs is one of the least understood areas. There are two principal ways in which competition works in voucher programs: among providers to join the program at the approval process (and to a lesser extent to remain in the program at contract renewal stage); and competition among providers to attract clients into their facilities. In areas where there are few providers (rural areas for instance), providers may compete ‘for the market’ as opposed to within the market, adapting their services to suit the needs of potential new clients who might otherwise not use a service at all.

Although more than half of the programs were judged by the authors to have strong competition between providers (defined as facilities contracted from more than one sector or sufficient facilities from a single sector that likely improved clients’ choice) this has not been monitored, either through external research or internal program monitoring. In theory, voucher programs with greater competition between providers should be more effective with lower prices and better quality. However, there are examples of functioning voucher programs where only public sector facilities are contracted and further investigation is needed to compare and contrast the role of competition between these types of program.

## Discussion

As we have seen, the defining features of a voucher program are: a governing body, a management agency or managing agency, contracted providers which receive a reimbursement payment according to services provided, and a target population. There are, however, many different configurations with regard to both the nature and type of governing body, management agency, providers, benefits and target population.

Despite these different configurations, all voucher programs provide incentives to consumers, who use the voucher and transfer the incentives to the provider. The ultimate objective is a change in behaviour, not only of the consumer but also of the provider and this does not differ between programs. The means of achieving such behaviour change is the incentive structure on both the demand- and supply-side, which is perhaps the most relevant structural feature of the voucher approach, overriding differences in design and implementation arrangements. In all programs reviewed a positive response is observed, with providers responding better to the clients and investing voucher revenue to make their facility more attractive, and consumers changing their health seeking behaviour to access services which they were not previously using.

The review illustrates that all voucher programs aim to increase utilization of a priority service; sometimes for the general population where underutilization is widespread, but mostly by poor or under-served groups where the uptake of critical services is lower than national or regional levels. The focus on the poor or other vulnerable groups is intended to stimulate care seeking by individuals, who in the absence of the voucher would likely have not sought care. There would be little value in giving subsidies to individuals who would use the facility services regardless.

Looking at the range of objectives of the voucher programs under review, it is clear that vouchers are successfully used to address tiered or multiple objectives, such as increased service utilization for a particular service or set of services, leveraging of private sector provision (in many cases to fill gaps in public provision), and targeting of a particular group, e.g. in Gujarat where the voucher scheme enables free access by poor women to private obstetricians for institutional delivery.

Unsurprisingly perhaps, the stated objectives of voucher schemes differ according to who initiates, finances and is responsible for them, and programs initiated by donors often have a wider health sector agenda, such as KfW's aim to encourage countries towards the introduction of social health insurance or USAID’s focus on vouchers as a gateway to building public private partnerships. In Cambodia, vouchers are used to extend the reach of the Health Equity Funds to lower levels of the health system (health center level) and are also used as a mechanism to verify that the client actually used the services [16].

It is also important to note that the majority of the programs (35 out of 40) use vouchers to enable contracting of the private sector. Vouchers make it feasible to contract private providers, because they enable provider registration, quality assurance, monitoring and tracking of payments. This is particularly relevant in the absence of national or sector-specific public private partnership (PPP) policies or strategies, and may provide a useful example to government (including at the local level) of contracting-out service provision. Where there is very little public service provision (e.g. Taiwan, Korea and Armenia), vouchers are or have been used both to channel subsidies to specific groups, and to control private providers (e.g. curb informal payments, which is an explicit objective of the Armenia Health Certificate Program). Importantly, vouchers provide a means of enabling governments to leverage the private sector capacity to meet public health goals such as the MDGs (i.e. Kenya, India and the newly scaled-up program in Uganda).

Most voucher programs seek to address the existing poor-rich inequities in access to life saving services, such as safe motherhood and family planning. While evidence is growing that vouchers can and do improve equity (in that they lead to greater increases in utilization among the poor than among the non-poor) this is an important area for further research. The capacity of vouchers to target a particular group is possibly the most important advantage when compared to other RBF approaches. In Asia where a majority of people, including the poor, finance their health care through out-of-pocket payments, usually in the private sector and even in the context of free public health service provision, this enables subsidies to follow those clients who most need them, thus helping to avoid catastrophic payments associated with certain high cost services such as Caesarean-sections for complicated deliveries.

Because voucher programs can be implemented using many different configurations, they can be adapted to local contexts and work under very different circumstances, while still producing the expected results. This flexibility makes vouchers highly suitable to apply in tandem with other RBF approaches, such as cash transfer programs and performance-based financing interventions. Furthermore, as we have seen, voucher distribution systems are used to channel non-financial benefits such as transport and food, as well as additional financial subsidies (i.e. cash payments, insurance benefits) to voucher clients.

The above analysis shows a significant increase in voucher programs from 2005 onwards (see Figure 3 above). This is largely made up of a proliferation of small schemes, either attached to social franchising networks where vouchers enable franchised providers to use subsidies (often from donors) to attract poorer clients, or small pilot schemes, introduced with a view to future scaling-up. Although they can provide useful learning, these pilot schemes can lead to fragmentation and do not always support the smooth progression towards universal health coverage which national governments seek to follow. The past decade has also witnessed the development of six larger voucher programs, not seen since the 1960s and 70s, at a rate of one new scheme every two years since 2006.

We can only speculate about the reasons for this increase, but from the review of 40 programs it is reasonable to hypothesise that a number of factors may be at play, including the capacity of vouchers to target the most vulnerable and contribute to their positive health seeking behaviour; and the flexibility of vouchers to work across a wide range of contexts, employing different configurations of organisations to oversee and implement the program, and different types of providers, benefits and target population groups. Disappointing progress towards achieving the MDGs (particularly MDG 4 and 5), together with increasing attention on achieving universal health coverage, has also led to more detailed analysis of who is left out in terms of access to critical public health services and, as we have seen, vouchers do offer a solution to targeting underserved groups. While donor financing for social franchising has been increasing, it brings with it an equity-related objective to reach the poor – hence the continued rise in the number of voucher schemes linked to franchises. And finally, the number of research papers on vouchers has increased significantly over the past five years [17] leading not only to higher visibility, but also increased confidence in the approach.

For those working in the design of voucher programs, there is increasing realisation that vouchers constitute a useful approach to enabling access, not only to SRH services but to other critical public health services such as chronic conditions and infectious diseases such as TB. It is likely that we will see a wider basket of voucher services over the next decade. As can be seen in Tanzania, as well as introducing many of the key capacities for social health insurance, vouchers are used to provide access to existing insurance schemes, and in high income countries there are voucher programs that provide access for specific target groups, such as immigrant workers in the US, or older people in Hong Kong, to a wider spectrum of services. A continuum would seem to exist with simple voucher schemes providing access to a single service for a specific disadvantaged group at one end, and social health insurance at the other. Voucher schemes which provide access to a wider basket of services and where a nominal fee is charged for the voucher at the distribution point (pre-payment) are closer to insurance programs on this continuum.

### Criteria for successful voucher programs and their limitations

However, not every health intervention lends itself well to the voucher approach. Using the extensive review of the literature and authors' own experiences, the following criteria were developed for successful voucher program design:

1. Voucher services should relate to a common condition in order to ensure sufficient demand which in turn ensures sufficient client volume for participating providers, and facilitates the identification of, and voucher distribution to, eligible people;

2. Services need to be clearly definable in order to allow for manageable claims processing, i.e. conditions for which the clinical need is largely predictable, with clearly defined criteria for diagnosis and disease severity, agreed protocols for management, as well as tightly defined management protocols that are common across groups of consumers;

3. Services should have a start and an end to limit payment to clear conditions, i.e. pregnancy and delivery, or tuberculosis diagnosis and treatment. With child care or even chronic conditions, this can be done by defining the length of time during which the treatment is covered by the voucher, and after which the patient is appropriately referred to the corresponding services or ceases to receive the subsidy;

4. Acute cases cannot be addressed through this approach because the patient needs time to learn of and understand the scheme, receive and use the voucher. Accidents or other sudden conditions, such as gender based violence (GBV), are not ideally suited to vouchers, as illustrated by the slow up-take of the GBVR vouchers in Kenya. For these schemes to work, the vouchers must be kept at the health facility and knowledge must be widespread in the surrounding communities that services are available and provided for free. In such cases, vouchers act predominantly as a reimbursement mechanism;

5. The treatment for the intervention should be offered (or potentially offered) by a range of providers. Special treatments that can only be offered in referral hospitals do not suit this approach unless the treatment is a referral from a voucher service provided at lower level facilities (such as a Caesarean-section or treatment of cervical cancer);

6. To justify the costs of voucher distribution and claims processing, interventions should be priority services as defined by the Ministry of Health, which are currently under-consumed by a specific group, and relevant to solve important public health problems (e.g. high maternal mortality, unsafe abortion, high fertility, high health system costs of untreated diabetes and so on).

7. There are additional aspects of voucher programs’ performance that cannot be discussed in this review as the data are simply not available. However, going forward, voucher programs could make substantial contributions by adapting standard performance indicators. The five here, framed as questions, could help to measure and compare the performance of voucher interventions using routine program data:

● What proportion of clients are new users/adapters in any given voucher service?

● How cost effective or efficient is any given voucher service?

● What is the level of quality in any given voucher service?

● How many DALYs averted or CYPs gained can be attributed to any given voucher service (net programmatic contribution)?

● What proportion of voucher clients are poor in any given voucher service?

All services currently provided through the voucher schemes identified in this review adhere to these seven criteria set out above.

## Conclusion

Vouchers are a promising and increasingly visible approach to target subsidies to individuals who, in the absence of the subsidy, would likely not have sought care. A large increase has been observed in the number of voucher programs since 2005, and a corresponding increase in the number of studies of voucher programs, contributing to building the evidence base. Possible reasons for this are related to specific strengths of voucher schemes, such as the ability to incentivise changes in behaviour among both consumers and providers (a combined demand- and supply-side effect), and the capacity to target and channel resources to the most vulnerable. Other more political factors, such as the increasing attention paid to achieving universal health coverage and donor interest in reducing inequities in access to essential health services have also played a part.

Whereas the review identified many differences between voucher schemes related to their structure and implementation arrangements, the commonalities, and in particular the incentive structure whereby the client passes the subsidy or incentive to the provider, seem to override many of these differences. In all programs, a positive behavioural response is observed with providers investing voucher revenue to improve quality and attract more clients, and clients accessing SRH services which they were not previously using.

The review found that all programs aim to increase utilization of priority health services, particularly SRH services, mostly among poor underserved and/or vulnerable populations. The programs reviewed adhere to criteria that make voucher services more functional, such as services that are related to relatively common conditions, are clearly definable, time-limited (with a beginning and an end) and are sufficiently relevant within the country’s health policy framework to justify the costs of voucher distribution and claims processing. New voucher programs are already providing access to a wider basket of voucher services and it is likely that this trend will continue over the next decade, with vouchers for child health, chronic conditions and infectious diseases.

The review also noted that, while most voucher programs remain too small to adequately address national level need among the poor, large programs are being developed at a rate of one program every two years since 2006, with further programs in the pipeline. The importance of addressing the huge poor-rich inequalities in access to basic services is well recognized as an important component in the drive to achieve universal health coverage; vouchers are increasingly acknowledged as a promising targeting mechanism in this context. As the movement for universal coverage develops [18], greater consideration could be given to the relative strengths and weaknesses of targeted social protection programs and the role of vouchers in achieving larger equity gains in the health sector, especially in contexts where a full social health insurance model is not cost-effective.

## Endnotes

^a^Most countries where a program was identified had a Gross National Income (GNI) below US$ 1,400 in 2010 with the exception of Armenia (3,200 US$). The GNI of China, Korea, Indonesia and Taiwan was also higher than US$ 1,400 in 2010, but at the time of implementation this was much lower. GNI per capita of low-income country in 2010 is less than 1,006 US$; lower-middle income country 1,006 US$-3,975 US$, upper middle income: $3,976 - $12,275; high income, $12,276 or more (World Bank: http://data.worldbank.org/indicator/NY.GNP.PCAP.CD).

## Appendix

Complete list of published documents consulted for the literature review, organised by country

General references

● Bellows N, Bellows B, Warren C: **The use of vouchers for reproductive health services in developing countries: systematic review**, 2011, *Trop Med Int Health*. Jan; **16**(1):84–96.

● Meyer C, Bellows N, Campbell M, Potts M: **The Impact of Vouchers on the Use and Quality of Health Goods and Services in Developing Countries: A systematic review** ***.*** 2011, London: EPPI-Centre, Social Science, Research Unit, Institute of Education, University of London. ISBN: 978-1-907345-10-4.

● Gorter AC and Bellows BW, **Do competitive voucher schemes improve the provision of health care to underserved and/or vulnerable population groups? Experiences from Nicaragua, India and Africa** ***.*** April 24 2008, Invited Seminar, Department of Social Medicine, University of Bristol.

● Sandiford P, Gorter A, Rojas Z, Salvetto M. **A guide to competitive vouchers in health** ***.*** Private Sector Advisory Unit, 2005, World Bank Group, Washington, DC, 2005. ISBN: 0-8213-5855-3.

● Gorter AC, Sandiford P, Rojas Z, Salvetto M. **Competitive Voucher Schemes for Health. Background Paper** ***.*** 2003, ICAS together with Private Sector Advisory Unit, World Bank Group, Washington, DC.

● Gorter A. **Evidence of effectiveness of competitive voucher schemes on HIV prevention and care for young people.** Background paper '*Global consultation on the health services response to the prevention and care of HIV/AIDS among young people*' organised by WHO with UNFPA, UNAIDS, YouthNet. Summary found in: *Achieving the global goals: access to services*, 17–21 March 2003, Technical Report of a WHO Consultation, Montreux, Switzerland.

Country specific references

ARMENIA

● Crape B, Demirchyan A, Grigoryan R, Martirosyan H, Petrosyan V, Truzyan N: **Evaluation of the Child Health State Certificate Program** ***,*** 2011, Center for Health Services Research and Development of the American University of Armenia, Yerevan, Armenia.

● Truzyan N, Grigoryan R, Avetisyan T, Crape B, Petrosyan V: **Protecting the right of women to affordable and quality health care in Armenia, analysis of the obstetric care state certificate program**, 2010, Center for Health Services Research and Development of the American University of Armenia, Yerevan, Armenia.

● **Obstetric Care State Certificate Program:** CHS Newsletter Issue 5, Winter-Spring 2010, American University of Armenia, Yerevan, Armenia.

● Avetisyan T: **Equitable cost burden for women: evaluation of the obstetric care state certificate program in Armenia**, 2011, Presentation School of Public Health, Boston University, Boston, USA.

BANGLADESH

● Hatt, L, Nguyen H, Sloan N, Miner S, Magvanjav O, Shrama A, Chowdury J, Chowdury R, Paul D, Islam M, Wang H: **Economic Evaluation of Demand-side Financing (DSF) Program Maternal Health in Bangladesh**. Bethesda, MD, Abt Associate Inc.; 2010.

● Schmidt, JO, T Ensor, Hossain A, Khan S: **Vouchers as demand side financing instruments for health care: A review of the Bangladesh maternal voucher scheme**. *Health Policy* 2010, **96**(2): 98–107.

● Ahmed S, Kahn MM: **A maternal health voucher scheme: what have we learned from the demand-side financing scheme in Bangladesh?***In Health Policy and Planning* 2010, 1–8, doi:10.1093/heapol/czq015

● Ahmed S, Kahn MM: **Is demand-side financing equity enhancing? Lessons from a maternal health voucher scheme in Bangladesh**. *Social Science and Medicine* 2011, **72**(10): 1704–10.

● Nguyen HTH, Hatt L, Islam M, Sloan NL, Chowdhury J, Schmidt JO, Hossain A, Wang H: **Encouraging Maternal Health Service Utilization: An Evaluation of the Bangladesh Voucher Program.***Social Science and Medicine* 2012, 74(7): 989–96.

● Rahman, M, Rob U, Tasnima K: **Implementation of the Maternal Health financial Scheme in Rural Bangladesh.** Population Council; 2009.

● Rob U, Rahman R, and Bellows B: **Using vouchers to increase access to maternal health care in Bangladesh**. *International Quarterly of Community Health Education* 2010, **30**(4): 293–304.

● Koehlmoos T.L.P., A. Ashraf, et al.: **Rapid assess-ment of demand-side financing experiences in Bangladesh** ***.*** Dhaka, International Centre for Diar-rhoeal Disease Research, Bangladesh, 2008.

● GTZ HNPSP Team, ICDDR,B and Data International; Ministry of Health and Family Welfare: **Report on Rapid Assessment of Demand Side Financing (DSF) Pilot**, October 2008, Research Paper 35.

● Ahmed S, **Effect of maternal health vouchers on access to healthcare services in Bangladesh: Testing the inverse-equity hypothesis**. International Health Economics Association Abstract, July, 2011 [abstract].

CAMBODIA

● Ir P, Horemans D, Souk N, van Damme W: **Using targeted vouchers and health equity funds to improve access to skilled birth attendants for poor women: a case study in three rural health districts in Cambodia.***BMC Pregnancy and Childbirth* 2010, **10**: 1–11.

● Ir P, and Wilkinson D: **Social Health Protection for the Poor and Vulnerable in Cambodia: the Role of Vouchers**. April 2011, Consultancy Report.

● Cambodia Ministry of Health and KfW ***: *** **Social Health Protection Program Vouchers for Reproductive Health Services**. 2011, Quarter 2 Report. EPOS Health Management.

● Marie Stopes International Cambodia: **A pilot results-based approach to increasing access to quality reproductive health services for peri-urban and rural populations in Cambodia,** 2011, Phnom Penh, Cambodia.

● Gorter AC: **Quick Assessment of Vouchers implemented by MSIC in Cambodia** Workshop building OBA capacity MSIC staff: March 2011. Project “*Building Capacity in Local Authority and Private Sector Sexual and Reproductive Healthcare Providers in Viet Nam and Cambodia*”, EU project implemented by Marie Stopes, Cambodia.

● Reproductive Health Association of Cambodia: **Health Equity Fund with Reproductive Health Focus Project***,* 2011, Final Report.

● Sovannarith E, Keovathanak K**: Review of Health Equity Funds for Reproductive Health in 5 Operational Districts,** 2010, unpublished report.

● Hiwasa A. **The Experiences and Perspectives on Birth Preparedness from Women and Com-munities in Rural Cambodia: Rethinking the “The First Delay”,** A thesis submitted to a partial fulfillment of the requirements of the Master of Science Programs, The Royal Tropical Institute, Amsterdam, The Netherland, August 2010.

● Souk N, Horemans D, Ir P: **Follow-up Evaluation of Voucher Scheme for Safe Delivery in Three Operational Health Districts in Kampong Cham**; Project *Provision of Basic Health Services in the Provinces of Siem Reap, Otdar Meanchey and Kampong Cham* (PBHS2), October, 2010, Cambodia.

● Ir P, Horemans D, Souk N, van Damme W, **Improving access to safe delivery for poor pregnant women: a case study of Vouchers plus Health Equity Funds in three health districts in Cambodia**; *Studies in Health Services Organization & Policy*, 24, 2008.

● IGES Institut GmbH: **Linking Directly Targeted Subsidies for Health Care Provision with Health Equity Funds & Social Health Insurance Feasibility Study about a Voucher Scheme for Safe Delivery and Family Planning Services in Cambodia**, 2008.

● **Quarter 2 Report** by EPOS Health Management, Cambodia Ministry of Health & Kreditanstalt für Wiederaufbau (KfW), In the *Social Health Protection Programme - Vouchers for Reproductive Health Services*; January 2011

● Murakami H, Nagai M, Matsuoka S, Obara H: **Performance-based Financing of Maternal and Child Health Services: Financial and Behavioral Impacts at the Field Level in Kampong Cham Province**; in JICA Project *Improving Maternal and Child Health Services in Rural Areas in Cambodia*”, February 2009, Cambodia.

CHINA

● Du K, Zhang K, Tang S: **Draft report on MCHPAF study in China**. World Bank, Washington, D.C., 2001.

● Bloom G, Liu J, Qiao J, **A Partnership for Health in China Reflections on the Partnership Between the Government of China, the World Bank and DFID in the China Basic Health Services Project** ***.*** Practice Paper Volume 2009 Number 2. Institute of Development Studies May, 2009, UK.

● Gwatkin D, **Inequalities in Access to Health Care in Developing Countries: What Should Be Done?** Health/nutrition/population (HNP) and poverty seminar report, March 21, 2001.

● Huntingdon D, Yunguo L, Ollier L, Bloom G: **Improving maternal health – lessons from the basic health services project in China**. DFID Briefing January 2008.

HONG KONG

● Yam CHK, Liu S, Huang OHY, Yeoh E, Griffiths SM: **Can vouchers make a difference to the use of private primary care services by older people? Experience from the healthcare reform programme in Hong Kong**. *BMC Health Services Research* 2011, **11**:255.

● Department of Health, **Health Care Voucher Scheme for the Elderly**, September 2, 2008, Hong Kong.

INDIA – AGRA (USAID)

● Mishra AK, Singh S, Sharma S, Sharma S, Singh S, Dixit M, Ja S**: Does Demand Side Financing Help in Better Utilization of Family Planning & Maternal & Child Health Services?** Evidence from Rural Uttar Pradesh, India, International Conference on Family Planning Nov 29 –Dec 2, 2011, Dakar, Senegal.

● Das U, Ranjan R, Mishra A, Liberhan T, Kandwal A: **Scaling up of Vouchers: Improving Access, Equity, and Quality** ***,*** Futures Group International, International Conference on Family Planning Nov 29 –Dec 2, 2011, Dakar, Senegal.

● Arora R, Neogi S, Misra M: **Innovative Ways to Meet Health Challenges of Urban India A White Paper** ***,*** 2011, Public health Foundation of India, India.

● Donaldson, D., H. Sethi, and S. Sharma: **Vouchers to Improve Access by the Poor to Reproductive Health Services: Design and Early Implementation Experience of a Pilot Voucher Scheme in Agra District, Uttar Pradesh, India**. 2008, USAID Health Policy Initiative, Task Order 1, Futures Group International, Washington, DC.

● Riggs-Perla J, Bhattacharjee A, Quigley P, Raman AV, Harbison S and Karra M, **IFPS II Evaluation** ***,*** USAID. Report prepared through the Global Health Technical Assistance Project, September 2007, The Global Health Technical Assistance Project, Washington DC.

● Krishna S ***: *** **Vouchers for Equity in Health** ***.*** Presentation made in April 12–13, 2007 I Best Western Resort Country Club I Gurgaon, India

● **Public Private Partnership models in Health implemented in Uttar Pradesh**. Presented during NRHM Workshop on Sharing Good Practices in Partnerships with Non-governmental Sector for Public Health Goals, 2008, India.

● Shuvi S: **Shaping Demand and Practices to Improve Reproductive, Maternal, Newborn and Child Health and Nutrition Outcomes in Northern India: A Landscaping Grant Progress Report,** Futures Group Presentation on Social Franchising In India. November, 2008, June 1, 2009 – July 31, 2009.

● **Concurrent Assessment of Janani Suraksha Yojana (JSY) Scheme In Selected States Of India,** UNFPA, 2008, India.

INDIA GUJARAT

● Bhat R, Mavalankar DV, Singh PV, Singh N: **Maternal healthcare financing: Gujarat's Chiranjeevi Scheme and its beneficiaries.** Journal of Health, *Population, and Nutrition 2009*, **27**(2): 249–258.

● Mavalankar D, Singh A, Patel SR, Desai A, Singh PV: **Saving mothers and newborns through an innovative partnership with private sector obstetricians: Chiranjeevi scheme of Gujarat, India**. *International Journal of Gynecology and Obstetrics* 2009, **107**; 271–276.

● Bhat R, Singh A, Maheshwari S, Saha S. **Maternal Health Financing - Issues and Options: A Study of Chiranjeevi Yojana in Gujarat** ***.*** 2006, Working Paper, Indian Institute of Management Ahmedabad, India.

● Bhat R, et al. Maternal Healthcare Financing: Gujarat’s Chiranjeevi Scheme and Its Beneficiaries; *J Health Popul Nutr* 2009 April; 27(2):249–258.

● Singh PV, **Managing Maternal Health Care Services through Public Private Partnerships: Policy Issues and Implications** ***. From*** *A Study of the Chiranjeevi Scheme in Panchmahals District of Gujarat, India*, Thesis Submitted in Partial Fulfillment of the Requirements for the Fellow Program in Management Indian Institute of Management Ahmedabad.

● Acharya A and McNamee P**: Assessing Gujarat’s ‘Chiranjeevi’ Scheme**. In *Economic & Political Weekly,* EPW November 28, 2009 vol XLIV no 48.

● Bhat R et al.: **Maternal Health Financing in Gujarat: Preliminary Results from a Household Survey of Beneficiaries under Chiranjeevi Scheme** ***.*** 2007, Indian Institute of Management Ahmedabad, India.

● Krupp K and Madhivanan P**: Leveraging human capital to reduce maternal mortality in India: enhanced public health system or public-private partnership?** In *Human Resources for Health* 2009, **7**:18

● McNamee P and Acharya A: **Public-private partnerships to reduce maternal mortality: silver bullet or smoking gun?** [http://iussp2009.princeton.edu/papers/93495] (Accessed September 21 2009).

● **Public-Private Partnerships: Managing contracting arrangements to strengthen the reproductive and child health programs in Indian - Lessons and Implications from three case studies**, 2007, Indian Institute of Management Ahmedabad, India.

● **Rapid Assessment of Chiranjivee Yojana in Gujarat** 2006, India.

● **Chiranjeevi Yojana (Plan for a long life): Public-private partnership to reduce maternal deaths in Gujarat**, India. [http://www.unicef.org/devpro/46000_47108.html]

INDIA MAMTA DELHI

● **Evaluation of Mamta Scheme in National Capital Territory of Delhi,** Department of Planning & Evaluation, National Institute of Health and Family Welfare., Report January 2010, India.

INDIA HARYANA

● **District Health Action Plan 2010–2011** ***,*** National Rural Health Mission. District Health Society West-Champaran, Bihar

● Janani Suvidha Yojna, **Promoting institutional deliveries in the urban slums initially in eight districts of Haryana**, India.

INDIA KOLKATA

● **Private Health Insurance in India: promise and reality** ***,*** ILO report prepared by BearingPoint, Inc. for USAID. February 2008.

● Gupta I, Joe W, Rudra S. **Demand Side Financing in Health: How far can it address the issue of low utilization in developing countries?** 2010, World Health Report, Background Paper 27, WHO, Geneva, Swiss.

INDONESIA

● Daly P and Saadah F: **Indonesia: Facing the Challenge to Reduce Maternal Mortality**. East Asia and the Pacific Region Watching Brief, June 1999 Issue 3.

● Knowles JC: **Consultant’s Report of Technical Assistance provided to the BDD Sustainability Component of the Safe Motherhood Project** (15–26 May, 2000) Draft: 14.6.00, Indonesia.

● **Making Services Work for the Poor: Nine Case Studies from Indonesia** ***;*** 2006, Indonesia Poverty Analysis Program (INDOPOV), Poverty Reduction and Economic Management Unit East Asia and Pacific Region, World Bank.

● Tan ESM**: Making services work for the poor in Indonesia case study 2: vouchers for midwife services in Pemalang district, central Java province** ***;*** The World Bank April 2005.

● Gwatkin DR, Wagstaff A, and Yasbeck A ***: *** **Reaching The Poor with Health, Nutrition, and Population Services: What Works, What Doesn’t, and Why**, 2005, World Bank, Washington, DC.

KENYA

● Bellows B, Kyobutungi C, Mutua MK, Warren C, Ezeh A: **Increase in facility-based deliveries associated with a maternal health voucher program in informal settlements in Nairobi, Kenya**. In *Health Policy and Planning* 2012, Mar 21.

● Lenel A and Griffith D: **Voucher schemes as a financing option in the health sector - the experience of German Financial Cooperation** ***.*** Working Paper, September 2007, Germany.

● Gorter A and Aida B ***: *** **Midterm review of the *‘*** **Development of the Health Sector (Swap) Program - Reproductive Health Voucher Scheme (Output Based Approach)-*Kenya’****, 2011,* National Coordination Agency for Population and Development and KfW, implemented by EPOS Health Management, Bad-Homburg, Germany.

● Bellows B, Hamilton M, Kundu F (2009 ***): *** **Vouchers for Health: Increasing utilization of facility-based FP and safe motherhood services in Kenya***.* Bethesda, MD: Health Systems 20/20 project, Abt Associates Inc.

● Patsika R, et al.:**Promoting Family Planning Use in Kenya through Output-Based Aid: Strategic Marketing Recommendations to NCAPD and KfW**. 2009, Bethesda, MD: Private Sector Partnerships-One project, AbtAssociates Inc.

● Morgan L ***: *** **More Choices for Women: Vouchers for Reproductive Health Services in Kenya and Uganda** ***.*** 2011, World Bank, Washington, DC.

● Janisch C.P, Albrecht M, Wolfschuetz A, Kundu F. Klein S**: Vouchers for health: A demand side output-based aid approach to reproductive health services in Kenya**; 2010, In *Global Public Health, An International Journal for Research, Policy and Practice***5**:6, 578–594.

● Mati JKG et al: **Report of the Mid-Term Review of the Reproductive Health – Output-Based Approach Project In Kisumu, Kitui, Kiambu, Korogocho And Viwandani 2005–2008** ***,*** 2008, submitted to NCAPD, Nairobi, Kenya.

● Population Council ***: *** **The reproductive health vouchers program in Kenya – Summary of findings from program evaluation***,* 2011, Reproductive Health Vouchers Evaluation Team of PopCouncil, Nairobi, Kenya.

● Aneesa A, Gitonga N, O’Hanlon B, Kundu F, Senkaali M, Ssemujju R:. **Insights from Innovations: Lessons from Designing and Implementing Family Planning and Reproductive Health Voucher Programs in Kenya and Uganda**. November 2009, Bethesda, MD: Private Sector Partnerships-One project, Abt Associates Inc.

● Kilonzo M, Senauer K, Switlick-Prose K, Eichler R ***: *** **Paying for Performance: The Reproductive Output Based Aid Program in Kenya** ***.*** 2010, P4P Case Studies, Health Systems 20/20, Bethesda, MD, Abt Associates Inc.

● Erulkar AS, **Behavior Change Evaluation of a Culturally Consistent Reproductive Health Program for Young Kenyans**; *International Family Planning Perspectives*, 2004, **30**(2):58–67

● Ochieng B et al.:**Friends of the Youth - A youth-adult HIV/AIDS behavior change program for urban Kenyan youth**. 2007, Program Brief, Population Council, Nairobi, Kenya.

KOREA

● Ross JA, Han DW, Keeny SM, Cernada GP, Hsu TC, Sun TH**: Korea/Taiwan 1969. Report on the National Family Planning programs**, 1970 *Studies in Family Planning*, Vol 1, No 54, 1–16.

● Taek Il Kim., Ross JA., Worth GC ***: *** **The Korean National Family Planning Program, Population Control and Fertility Decline** ***,*** 1972, Population Council, New York, USA.

● Ktsanes V: Review of article by *Taek Il Kim., Ross JA,. Worth GC. The Korean National Family Planning Program: Population Control and Fertility Decline*, American Anthropologist, New Series, Vol. 75, No. 4. (August 1973), p. 1150.

● Stoeckel J**: Differentials in Fertility, Family Practice, and Family Size Values in South Korea, 1965–1971**, 1975, *Studies in Family Planning*, Vol 6, No 11, 378–401.

● Hong SB ***: *** **Korea, a Government Program employing Private Physicians and Fieldworkers, in Surgical FP methods, the role of the private physician** ***,*** 1981, International Fertility Research program, Research Triangle Park, North Carolina, USA.

● Cho, Nam Hoon., Sae Kwon Kong, and Jong Kwon Lim. 1984. **Recent Changes in ContraceptiveUse and Fertility in Korea**. In *Journal of Population and Health Studies***4**(2):63–79.

● Robey, B: **Community-based Contraceptive Distribution: A Korean Success Story, 1987,***Asia-Pacific Population & Policy* (4).

● Chung SH **Determinants of fertility control in Korea,** 1990, *Korea J Popul. Dev.* July **19**(1): 27–46

● Cho, Nam Hoon, Moon Hee Seo, and Boon Ann Tan: **Recent Changes in the PopulationControl Policy and Its Future Directions in Korea**, 1990, *Journal of Population, Health and Social Welfare***10**(2):152–172.

● Cho, Nam Hoon, and Hyun Oak Kim. 1992. **An Overview of the National Family PlanningProgram in Korea: A Summary Explanation**. Seoul: Korea Institute for Health and Social Affairs, 19–26.

● Ross, John A., John Stover, and Demi Adelaja. 2005. **Profiles for Family Planning and ReproductiveHealth Programs: 116 Countries***,* 2nd ed. Glastonbury, CT: Futures Group.

● Taek Il Kim and John A Ross (2007), **The Korean Breakthrough**, in Warren C. Robinson and John A. Ross (editors), *The global family planning revolution: three decades of population policies and programs*, 2007,World Bank, Washington D.C.

MADAGASCAR

● Marie Stopes International: **Using mobile finance to reimburse sexual and reproductive health vouchers in Madagascar** ***,*** 2011, London, UK.

● Plautz A et al. **The Impact of the Madagascar TOP Réseau Social Marketing Program on Sexual Behavior and Use of Reproductive Health Services**. 2003, PSI Research Division Working Paper No. 57 2003.

● **Franchised Youth Clinics Motivate Behavior Change in Madagascar**, PSI Research Brief No. 4, August 2004

● **Clinical Social Franchising Compendium: An Annual Survey of Programs**, 2010, The Global Health Group: University of California, San Francisco.

MYANMAR

● The Global Health Group (2010), **Clinical Social Franchising, Case Study Series, Sun Quality health, Population Services International/Myanmar** ***,*** University of California, San Francisco.

● USAID and SHOPS (2010). Private Sector Working Group Meeting, February 3, 2010, *Meeting Minutes*.

NICARAGUA (Sex Workers)

● Gorter AC, McKay J, Meuwissen L, Segura Z, Medina J and Bellows B, **Targeting vouchers to underserved populations in Nicaragua,** oral presentation in panel: "Vouchers for Health", The Global Health Council's 36th Annual International Conference on Global Health, May 26–30, 2009, Washington, DC.

● Gorter AC, Segura ZE, Medina JA, Rodriguez OG, Medina GM, Peralta WJ and Rovin K, **Providing STI/HIV/AIDS services to glue-sniffing young people in Nicaragua: needs, relevance and feasibility***,* XVII International Conference on AIDS, 3–8 August 2008, Mexico City.

● Gorter AC, Segura ZE, Savelkoul PHM, Morré SA ***: *** **Chlamydia trachomatis infections in Nicaragua: Preliminary results from a competitive voucher scheme to prevent and treat sexually transmitted infections and HIV/AIDS among sex workers** ***,*** In *Drugs of Today* 2006, 42 (Suppl. A): 47–54.

● McKay JE, Campbell DJ, Gorter AC. **Lessons for management of STI treatment programs as part of HIV/AIDS prevention strategies**. *American Journal of Public Health* 2006; **96**:7–9.

● Borghi J, Gorter A, Sandiford P and Segura Z. **The Cost-Effectiveness of a Voucher Scheme to Reduce Sexually Transmitted Infections in High Risk Groups: the case of Managua, Nicaragu**a. In *Health Policy & Planning,* 2005; **20**(4): 222–31.

● Gorter AC, Segura ZE, Medina JA, McKay JE. **Effectiveness and impact of a long running competitive voucher program providing quality STI/HIV care to groups most at risk of HIV in Nicaragua.** Poster XVI International Conference on AIDS, Toronto, Canada, 13–18 August 2006.

● Gorter A, Segura Z, Sandiford P, Zuñiga E, Torrentes R, Ådahl S: **Involving partners and regular clients of sex workers in a voucher programme for improved medical care in Managua**. Research for Sex Work Newsletter 3, July 2000, Amsterdam.

● Sandiford P, Salvetto M, Segura Z, Gorter A: **Clinics for sex workers in Managua.** In Harper M (ed.) *Public services through private enterprise; micro-privatisation for improved delivery*, IT Publications, London, and Oxford IBH Publishers New Delhi, 2000.

● Gorter A, Sandiford P, Segura Z, Villabella C: **Improved health care for sex workers: a voucher program for female sex workers in Nicaragua.** Research for Sex Work, No. 2, August 1999, pp. 11–13.

NICARAGUA (adolescents)

● Meuwissen LE, Gorter AC, Segura Z, Kester ADM, Knottnerus JA: **Uncovering and responding to needs for sexual and reproductive health care among poor urban female adolescents in Nicaragua**. *Tropical Medicine and International Health* 2006, **11**(12), 1858–1867.

● Meuwissen LE., Gorter AC., Kester ADM and Knottnereus A**: Does a competitive voucher program for adolescents improve the quality of reproductive health care? A simulated patient study in Latin America.***BMC Public Health* 2006, 6:204. Published online August 7 doi:10.1186/1471-2458-6-204.

● Meuwissen LE., Gorter AC., Kester ADM and Knottnerus JA**: Can a comprehensive voucher program prompt changes in doctors’ knowledge, attitudes, and practices related to sexual and reproductive health care for adolescents? A case study from Latin America**. In *Tropical Medicine and International Health* 2006;**11**(6):889–898.

● Meuwissen LE., Gorter AC., Knottnereus A. **Impact of accessible sexual and reproductive health care on poor and underserved adolescents in Managua, Nicaragua: A quasi-experimental intervention study**. In *Journal of Adolescent Health*, 2006; **38**(1):56.

● Meuwissen LE, Gorter AC, Knottnereus A: **Perceived quality of reproductive care for girls in a competitive voucher program. A quasi-experimental intervention study**, Managua, Nicaragua. In *International Journal for Quality in Health Care*, 2006;**18**(1):35–42 (Epub 2006 Jan 18).

● Meuwissen L: **Improving sexual and reproductive health care for poor and underserved girls. Impact of a voucher program on access and quality of primary care in Nicaragua**. University of Maastricht, 2006. PhD dissertation.

NICARAGUA (Cervical Cancer)

● Salvetto M, Alvarado V**: A voucher scheme approach to screening for cervical cancer: the Nicaraguan experience,** In *Cancer Research Journal* 2008, **2** (2/3): 137–158.

● Howe SL, Vargas DE., Granada D., Smith JK. **Cervical cancer prevention in remote rural Nicaragua: A program evaluation**. In *Gynaecologic Oncology*, 2005; **99**: S232 – S235.

● Salvetto M and Sandiford P: **External Quality Assurance for cervical cytology in developing countries**, In *Acta Cytologica*, 2004 Jan-Feb, **48**(1):23–31.

● Lindsey H:**Researchers aiming to improve cervical cancer screening in developing countries**, *Oncology Times*, Part1 in Volume XXVI, No 9:page 22 and 27, 2004 and Part2 in Volume XXVI, No 10:page 42–4, 2004.

● Platero E**: Evaluación Final del Proyecto, prevencion del cancer cervico-uterino en mujeres pobres del area rural de los departamentos de Cuscatlan, La Paz y Morazan,** UNFPA, and DFID, El Salvador, 2004.

PAKISTAN (Greenstar)

● Agha S: **Impact of a maternal health voucher scheme on institutional delivery among low income women in Pakistan**. *Reproductive Health* 2011a, 8:10.

● Agha S: **Changes in the proportion of facility-based deliveries and related maternal health services among the poor in rural Jhang, Pakistan: results from a demand-side financing intervention**. *International Journal for Equity in Health* 2011, b, **10**:57.

● Beith A, Eichler R, Brown E, Button D, Connor C, His N, Sanjana P, Switlick K and Wang H ***: *** **Pay for Performance (P4P) to Improve Maternal and ChildHealth in Developing Countries: Findings from an Online Survey**. 2009, Bethesda, MD: Health Systems 20/20 project, AbtAssociates Inc.

● Eichler R, Islam M, Beith A ***: *** **Performance-based Incentives, Primer for USAID Missions** ***,*** 2010, Bethesda, MD: Health Systems 20/20 project, AbtAssociates Inc.

● Hamid B, Kazmi S, Eichler R, Beith A, Brown E ***: *** **Pay for Performance: Improving Maternal Health Services in Pakistan** ***,*** 2009, Bethesda, MD: Health Systems 20/20 project, AbtAssociates Inc.

● USAID, Pakistan Initiative for Mothers and Newborns (PAIMAN): Annual Report (October 2008 to September 2009).

● Haris Ahmed (2009), Subsidizing Maternal Health Services Cost To Improve Utilization, Presentation.

● Agha S ***: *** **The context for and development of a voucher program in rural Pakistan. Green star Social Marketing Pakistan**, 2009, Presentation made at the Asia Pay for Performance Workshop, Cebu, Philippines, January 19–23, 2009.

● Saleh A and Agha S ***: *** **New Approaches to Demand-Side Financing: The Jhang Voucher Scheme** ***.*** 2011, Greenstar Social Marketing, Karachi, Pakistan.

PAKISTAN (MSI)

● Griffith D:**Support to the PPP to increase demand, access, choices and quality services of Family Planning & Reproductive Health for underserved and poor communities***, 2009,* Draft Report, Marie Stopes Society, Pakistan.

● Boler T and Harris L:**Reproductive Health Vouchers: from Promise to Practice***.* London: Marie Stopes International.

● Rahal S and Khan FK**: Case Study: “Suraj” - A Private Provider Partnership: Marie Stopes Society, Pakistan**, 2010, Marie Stopes International.

● Syed K A, Ghulam M, Mohsina B, Waqas H, Muhammad A, Jamshaid A, Aftab A. **Perspectives and practices of client, provider and marketing worker of an effective family planning social franchise intervention in rural Pakistan: qualitative enquiries** [abstract].

● Syed K A, Waqas H, Mohsina B, Muhammad A, Ghulam M, Wajahat H, Aftab A, Jamshaid A. **Evidence to innovate: Reproductive health social franchising through output-based aid Vouchers in the Rural Areas of Pakistan** [abstract].

SIERRA LEONE

● Boler T and Harris L (2010). **Reproductive health vouchers from promise to practice** ***,*** London, Marie Stopes International.

● MSI Operations Manual Reproductive Health Vouchers in Sierra Leone - “HEALTHY BABY” (Safe motherhood) and “HEALTHY LIFE” (Family Planning Services), Sierra Leone.

TAIWAN

● Ravenholt R T and Frederiksen H**: Numerator analysis of fertility patterns**, *Public Health Rep*. 1968 June; **83**(6): 449–457.

● Chang, M., Liu TH, Chow LP: **Study by Matching of the Demographic Impact of an IUD Program: A Preliminary Report**, 1969, *The Milbank Memorial Fund Quarterly***47**(2): 137–157.

● Cernada G. and Chow L.P: **The Coupon System**, *American Journal of Public Health* Vol 59, No. 12, December 1969.

● Cernada G. and Chow L.P: **The Coupon System** in *The Taiwan Family Planning Reader*. Edited by George P. Cernada. Chinese Center for International training in Family Planning. Taiwan, 1970. pp (147–166).

● Ross JA, Han DW, Keeny SM, Cernada GP, Hsu TC, Sun TH**: Korea/Taiwan 1969, Report on the National Family Planning programs**, *Studies in Family Planning*, 1973, Vol 1, No 54, 1–16

● Hermalin AI, Chow LP**: Motivational factors in IUD termination: data from the second Taiwan IUD follow-up survey**. *J Biosoc Sci*. 1971 Oct, **3**(4):351–75.

● Yen CH., Wang CM: **Taiwan.** Studies in family planning. 7. *Stud Family Planning,* 1973 May, **4**(5):118–23.

● Sun TH: **An intensive effort to reduce fertility in Taoyuan County, Taiwan**: 1973 intermediate report. *Ind Free China*. 1974 Aug;**42**(2):6–15.

● Ching-Ching, Cernada G**: Taiwan**, in *special issue on Family Planning Programs: World Review*, *Studies in Family Planning*, 1985, Vol. 6, No. 8, Aug

● J. S. Chen, I. H. Su, L. P. Chow (1975), IUD Reinsertion in Taichung: A Study of First and Later Segments, *Stud Family Plann*. Vol. 6, No. 9, Sep., 1975 pp. 338–344.

● Lin CC, Huang M, **Taiwan´s National Family Planning program, in Surgical FP methods, the role of the private physician***,* 1981, International Fertility Research program, Research Triangle Park, North Carolina, USA.

● Chang MC**: Determinants of fertility control in Taiwan: an application of the Easterlin framework**. *Jing Ji Lun Wen.* 1984 Mar, **12**(1):123–52.

● Sun TH**: Promotion of a family planning program: the Taiwan model**. *South African Journal of Demography,* 1987 Jul;1(1):32–42.

● Ming-Cheng C, Freedman R, Sun TH: **Trendsin Fertility, Family Size Preferences, and Family Planning Practice:Taiwan, 1961–85,** 1987, In *Studies in Family Planning*, Vol 6, **18**:320–337.

● Freedman R, Chang MC, Sun TH: **Taiwan's transition from high fertility to below-replacement levels**. 1994, *Studies in Family Planning*, Volume: 25, Issue: 6 Pt 1, Publisher: Population Council, Pages: 317–331.

● Freedman R ***: *** **Observing Taiwan’s Demographic Transition: A Memoir**, 1998, Research Report No. 98–426, Population Studies Center, University of Michigan.

● Sun TH**: Impact of FP program on contraceptive/ fertility behaviour in Taiwan**, 2001, *Journal of Population Studies*, 23, 50–92

● Noel MD: **Experience of Family Planning Program in Taiwan, and menopausal women’s attitude on FP program**, 2005, MSc Thesis Public Health, National Yang-Ming University, Taiwan

● Cernada G, Sun TH, Chang MC, Tsai JF: **Taiwan's Population and Family Planning Efforts: An Historical Perspective,***International Quarterly of Community Health Education*, Volume 27, Number 2/2006–2007, p 99–120.

UGANDA (KfW)

● Bellows B, Hamilton M, Bagenda F, Mulogo E: **Associations between Uganda output-based voucher facilities, utilization of clinic treatment of sexually transmitted infections (STIs) and syphilis prevalence.** Accepted for publication in BMC, 2012.

● **The reproductive health vouchers program in Uganda, Summary of findings from program evaluation, report.**; 2011, Population Council Nairobi, Kenya: Reproductive Health Vouchers Evaluation Team, Population Council.

● Griffith D ***: *** **HIV/AIDS Prevention, Output based Aid, Introducing voucher systems for health care provision**, 2004, Final Draft report, for the program Financial Co-operation between Germany and Uganda.

● **STI prevalance and behavioural survey in Mbarara, Kiruhura, Ibanda, Isingiru and Bushenyi Districts**, Venture strategies for Health and Development & Mbarara University of Science and Technology, Preliminary report, December 2006.

● Morgan L: **More Choices for Women: Vouchers for *Reproductive Health *** **Services in Kenya and Uganda,** 2011, World Bank, Washington, DC.

● Lowe RF and Bellows BW ***:*** **Output-Based Aid to Treat Sexually-Transmitted Infections in Southwestern Uganda, A Study of the Impact of the Program on Participating Clinics,** 2007, Venture Strategies for Health and Development, School of Public Health, University of California, Berkeley, USA.

● Ho MR, Owusu EK, Aoki PM ***: *** **Claim Mobile, Engaging Conflicting Stakeholder Requirements in Healthcare in Uganda***, 2009*, International Conference on Information and Communication Technologies and Development. Doha.

● Boler T and Harris, L. **Reproductive Health Vouchers: from Promise to Practice**. London: Marie Stopes International, 2010.

● Bellows BW: **Social and Structural Determinants of Prevalence and Treatment of Sexually Transmitted Infections in Southwestern Uganda** ***,*** 2009, PhD dissertation - University Of California, Berkeley

● Bellows B ***: *** **Evaluation of output-based aid (OBA) in Uganda: Impact of contracted facilities and social marketed vouchers on knowledge, utilization and prevalence of sexually transmitted infections (STIs) 2006–2007** ***, *** **Bushenyi, Ibanda, Isingiro, Kiruhura, and Mbarara Districts** ***.*** 2008, Venture Strategies for Health and Development, School of Public Health, University of California, Berkeley, USA.

● Bellows B, Hamilton M: **Vouchers for Health: Increasing Utilization of Facility-Based STI and Safe Motherhood Services in Uganda**. 2009, P4P Case Studies – Uganda, Bethesda, MD: Health Systems 20/20 project, Abt Associates Inc.

● John Bua, Impact of Voucher System on Access to MCH Services in Eastern Uganda. Uganda Abstract University scheme. Jul 2011 [abstract]

● **Evaluation Of Output-Based Aid (OBA) I Uganda: Impact Of Contracted Facilities And Social Marketed Vouchers on Knowledge, Utilization And Prevalence Of Sexually Transmitted Infections (STIs) 2006–2007,** Venture strategies for Health and Development & Mbarara University of Science and Technology, report, 2006.

UGANDA (Makerere University)

● Ekirapa-Kiracho E, Waiswa P, Rahman MH, Makumbi F, Kiwanuka N, Okui O, Rutebemberwa E, Bua J, Mutebi A, Nalwadda G, Serwadda D, Pariyo GW, Peters DH. **Increasing access to institutional deliveries using demand and supply side incentives: early results from a quasi-experimental study**. *BMC International Health and Human Rights* 2011, **11**(S1):S11

● **Mu-Jhu Twinning Programme: Increasing Access to Institutional Deliveries Using Demand and Supply Side Incentives,** Safe Deliveries Pilot Project Report, February 2011, Annual report, Makerere University, Uganda.

VIETNAM

● Minh PD, Thang NC, van der Velden T, Le Ngoc Bao**: Involvement of private sector in HIV/AIDS prevention in Vietnam – a public-private partnership (ppp) model: increase access to services of the most at risk population (MARPS)***.2010, In Journal of Science*, Hue University, No 61, Vietnam.

● Ngo Van Huu. **Can private sector increase access to STI services of the most at risk population (MARPS)**? Evidence from Vietnam. The International AIDS Conference 2010 Vienna [abstract].

## Abbreviations

BCC: Behaviour change communication; BPL: Below poverty line; CCT: Conditional cash transfer; DFID: UK Department for international development; DSF: Demand side financing; FP: Family planning; GBV: Gender based violence; GBVR: Gender based violence recovery; GoK: Government of Kenya; GPOBA: Global partnership on output-based aid; HEF: Health equity fund; KfW: German development bank; MDGs: Millennium development goals; MIS: Management information system; MOH: Ministry of health; MOMS: Ministry of medical services; MOPHS: Ministry of public health and sanitation; MSI: Marie stopes international; NGO: Non-governmental organisation; NHIF: National hospital insurance fund; OCSC: Armenian obstetric care state certificate; PBC: Performance-based contracting; PBF: Performance-based financing; PMTCT: Prevention of mother to child transmission; PMU: Program management unit; PPP: Public private partnership; PSI: Population services international; RBB: Results-based budgeting; RBF: Results-based financing; RH OBA: Reproductive health output-based Aid; RHVP: Reproductive health voucher program; SF: Social franchise; SFO: Social franchising organisation; SHI: Social health insurance; SMH: Safer motherhood; SRH: Sexual reproductive health; STI: Sexually transmitted infection; TB: Tuberculosis; USAID: United States agency for international development; VMA: Voucher management agency; VP: Voucher program.

## Competing interests

The authors declare that they have no competing interests.

## Authors’ contributions

CG participated in discussions on the research agenda, worked with AG on the literature review, acquisition of data through networking, analysis and interpretation of those data, and led the final drafting of the manuscript. AG made substantial contributions to conception and design, led the acquisition of data, analysis and interpretation of data, and made substantial contributions to drafting the manuscript. BB conceptualized the initial research agenda, reviewed the data collection templates, and made substantial contributions to drafting the manuscript. JO was involved in literature review, interpretation, drafting, organizing and overall revision of the manuscript. All authors read and approved the final manuscript.
