# Erratum: Lessons from sexual and reproductive health voucher program design and function: a comprehensive review

**DOI:** 10.1186/s12939-015-0139-1

**Published:** 2015-02-17

**Authors:** Corinne Grainger, Anna Gorter, Jerry Okal, Ben Bellows

**Affiliations:** Options Consultancy Services Ltd., Senior Technical Specialist, Devon House, 58 St Katharine’s Way, London, E1W 1LB UK; Instituto CentroAmericano de la Salud, Epidemiology, Managua, Nicaragua; Population Council, Ralph Bunche Rd, PO Box 17643-00500, Nairobi, Kenya

In the final version of our article entitled, " Lessons from sexual and reproductive health voucher program design and function: a comprehensive review" the authors inadvertently failed to acknowledge the role of International Union of Scientific Study of Population (IUSSP). IUSSP colleagues kindly reviewed early drafts of the manuscript presented at an IUSSP workshop in Bangkok August 2012 and had specifically requested that we recognize their feedback. We therefore feel obliged to acknowledge IUSSP.
